# DIDA: A curated and annotated digenic diseases database

**DOI:** 10.1093/nar/gkv1068

**Published:** 2015-10-19

**Authors:** Andrea M. Gazzo, Dorien Daneels, Elisa Cilia, Maryse Bonduelle, Marc Abramowicz, Sonia Van Dooren, Guillaume Smits, Tom Lenaerts

**Affiliations:** 1 Interuniversity Institute of Bioinformatics in Brussels, Boulevard du Triomphe CP 263, 1050 Brussels, Belgium; 2 MLG, Département d'Informatique, Université Libre de Bruxelles, Boulevard du Triomphe, CP 212, 1050 Brussels, Belgium; 3 Center for Medical Genetics, Reproduction and Genetics, Reproduction Genetics and Regenerative Medicine, Vrije Universiteit Brussel, UZ Brussel, Laarbeeklaan 101, 1090 Brussels, Belgium; 4 Center for Medical Genetics, Hôpital Erasme, Université Libre de Bruxelles, Route de Lennik 808, 1070 Brussels, Belgium; 5 Genetics, Hôpital Universitaire des Enfants Reine Fabiola, Université Libre de Bruxelles, Avenue JJ Crocq 15, 1020 Brussels, Belgium; 6 AI lab, Vakgroep Computerwetenschappen, Vrije Universiteit Brussel, Pleinlaan 2, 1050 Brussels, Belgium

## Abstract

DIDA (DIgenic diseases DAtabase) is a novel database that provides for the first time detailed information on genes and associated genetic variants involved in digenic diseases, the simplest form of oligogenic inheritance. The database is accessible via http://dida.ibsquare.be and currently includes 213 digenic combinations involved in 44 different digenic diseases. These combinations are composed of 364 distinct variants, which are distributed over 136 distinct genes. The web interface provides browsing and search functionalities, as well as documentation and help pages, general database statistics and references to the original publications from which the data have been collected. The possibility to submit novel digenic data to DIDA is also provided. Creating this new repository was essential as current databases do not allow one to retrieve detailed records regarding digenic combinations. Genes, variants, diseases and digenic combinations in DIDA are annotated with manually curated information and information mined from other online resources. Next to providing a unique resource for the development of new analysis methods, DIDA gives clinical and molecular geneticists a tool to find the most comprehensive information on the digenic nature of their diseases of interest.

## INTRODUCTION

Identifying disease-causing genes and mutations is a central challenge in the human genetics field (1–4). Genomic data, especially those of clinical relevance, are organized and stored in databases, with the aim of describing the molecular relationships between genes and phenotypes. One widely used example of such a database is the Online Mendelian Inheritance in Man or OMIM database (5), which provides a collection of human genes and genetic phenotypes. The advent of cheap and efficient massive parallel sequencing (MPS) techniques has led to a significant increase in the amount of genomic data. Further efforts have hence been made to organize this novel wealth of data in databases of genomic variations, as for instance dbSNP (6) or the Leiden Open source Variation Database (7), while more targeted information regarding presumed pathogenic genomic variants linked to diseases are organized in specific databases such as ClinVar (8), DECIPHER (9) or Human Gene Mutation Database (10).

The current databases report on the relationships between isolated variants and phenotypes, focusing on monogenic or Mendelian disorders (11). Clearly, diseases may be also associated with mutations in multiple genes, which are referred to as oligogenic (or polygenic) disorders (12). An increasing number of such cases are being reported (13–18). More importantly, it has been argued that many disorders classically considered monogenic may be better described by more complex inheritance mechanisms (19). Long QT syndrome (14) is just one example. Consequently, the time has arrived to bundle the current knowledge as this will allow genomic researchers to expand their focus to this more difficult class of oligo- and polygenic disorders.

Digenic inheritance is the simplest form of oligogenic inheritance for genetically complex diseases and has been defined by Schäffer (19) as follows: ‘Inheritance is digenic when the variant genotypes at two loci explain the phenotypes of some patients and their unaffected (or more mildly affected) relatives more clearly than the genotypes at one locus alone’ (Note that in this manuscript, and also in the database we present here, we replace ‘locus’ with ‘gene’). As it is not possible to retrieve detailed records regarding digenic combinations from existing biomedical databases there is a clear need to develop new tools and services focusing on the digenic inheritance model. For instance, the simplest information concerning the combinations of variants mapped on genes, responsible for the development of a digenic disease, is often not available. We therefore developed DIDA (DIgenic diseases DAtabase), a novel database that provides for the first time a manually curated collection of genes and associated variants involved in digenic diseases. The database focuses currently on single nucleotide variants (SNVs) and small insertions or deletions (indels), excluding large indels as observed in Rotor syndrome (20), copy number variations (CNVs) as in 22q11 Deletion Syndrome (21) and repeats as in Facioscapulohumeral Muscular Dystrophy type 2 (22). This initial exclusion is because DIDA was developed to construct predictive tools that can determine how different small-scale mutations contribute to the onset of a digenic disease. Future developments of DIDA and those tools will also incorporate the other modification types with a new appropriate reorganization of the database tables, making it suitable to also include large-scale mutations. Additionally, we only included those data for which the causal mutations have been identified, excluding those digenic results that were obtained only through statistical techniques such as genetic linkage analysis, as was done for Fuchs corneal dystrophy (23). Notwithstanding those current restrictions, DIDA incorporates digenic evidence for already 44 human diseases. We expect to see an increase of instances over the years as MPS becomes more established and analysis methods become more advanced. DIDA makes all this digenic information publicly available in one location, providing an important resource that may lead to the intensification of the research into the combinatorial nature of many diseases, even when those diseases were previously considered to be monogenic.

## DATA COLLECTION AND CURATION

The search for examples of human digenic inheritance started from the SNV and small indel data reported in (19), which includes 95 gene pairs linked to 108 scientific articles published before January 2013. We manually mined from the literature the following information: variant coordinates at the genomic, coding DNA (cDNA) and protein level, variant zygosity state, allelic state of the digenic combination, the names of both genes and the disease, the patient's clinical symptoms and phenotype, and finally the familial or functional evidence for digenic inheritance. All genomic coordinates were checked with Alamut Visual version 2.5 (Interactive Biosoftware, Rouen, France) in order to have the correct positions as they are present in the human reference assembly GRCh37/hg19. This software was also used to map the cDNA and protein changes to the gene's longest transcript when possible, otherwise the transcript mentioned in the original publication was used. Each variant present in DIDA is accompanied by the NCBI transcript identifier on which the coordinates were mapped. The cDNA and protein coordinates for all variants are reported following HGVS recommendations (24). For intronic nucleotides, either a plus or a minus sign was used to specify their position relative to the beginning or ending of the exon. With Phenomizer (25,26) the patient's clinical symptoms and phenotype were translated into the corresponding Human Phenotype Ontology (HPO) identifiers. Diseases were classified into general World Health Organization International Classification of Diseases (WHO ICD10) categories (27). Orphanet (28) was used to retrieve a standard disease name with the corresponding Orphanet identifier (http://www.orpha.net, accessed September 2015). Also all OMIM (5) disease terms and the ICD10 disease identifiers were retrieved through Orphanet. Each digenic combination was categorized into one of two effect classes: either ‘on/off’ where variant combinations in both genes are required to develop the disease or ‘severity’ where variants in one gene are enough to develop the disease and carrying variant combinations in two genes increases the severity or affects its age of onset.

In total, 125 digenic combinations from 54 publications described in (19) were added to the database. A new PubMed search was conducted to include all relevant publications until June 2015. This led to the inclusion of 88 additional digenic combinations described in 28 unique publications. Pubmed references to all included publications are provided in the database (http://dida.ibsquare.be/references/).

## GENES, VARIANTS AND DIGENIC COMBINATIONS ANNOTATION

The manually collected data were enriched with annotations retrieved automatically from public databases. The purpose of this extra information is to assist in the creation of predictive models for digenic diseases, with the aim of understanding the genetic architecture behind these diseases. At the level of genes, we used gene symbols approved by the Human Genome Organization (HUGO) gene nomenclature committee (HGNC) (29). For each gene, the following information is available: the chromosome on which the gene is located, the correct Uniprot Accession number related to the protein of interest, the pathway in which the gene is implicated (from KEGG (30) and Reactome (31)), the motifs and functional domains conserved along the protein sequence (from Pfam (32) and Interpro (33)), the description of the function of the gene (from Uniprot (34)), the genes with which the gene of interest interacts (from IntAct (35), BIOGRID (36) and ConsensusPathDB (37)) and the tissues and/or organs in which the gene is expressed (GNF/Atlas (38)). Furthermore four features regarding the importance of the gene and its allelic state were added: the estimated probability of haploinsufficiency of the gene (39), the estimated probability that the gene is a recessive disease gene (40), the known recessive status of the gene (40) and the known essential status of the gene based on the Mouse Genome Informatics database (41). For each (missense) variant, the following extra information is available: the effect of the variant at the amino acid level, the pathogenicity predictions for PolyPhen2 (42) and SIFT (43), the unique id for dbSNP version 141 (6), the variant allele frequency for all individuals sequenced in phase 1 of the 1000 genomes project (44) and for all sequenced European- and African-American individuals of the Exome Sequencing Project (ESP6500) (45) (http://evs.gs.washington.edu/EVS/, accessed September 2015). These annotations were retrieved through dbNSFP v.2.8 (46).

## DATABASE DESCRIPTION

**Figure 1.**
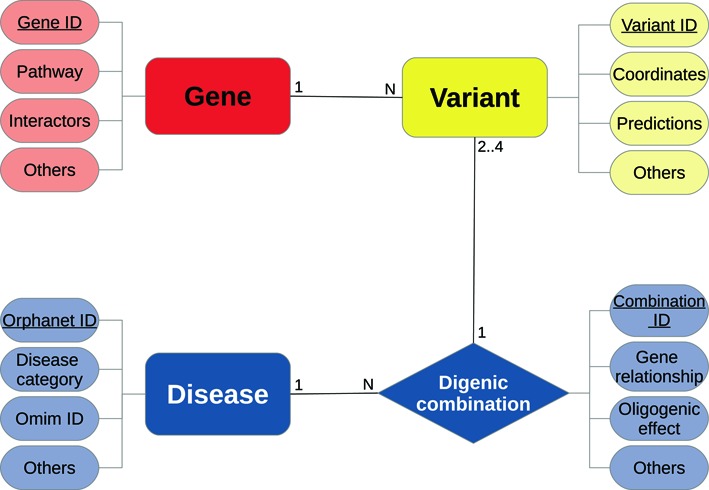
DIDA entity relationship diagram. The main entity is the digenic combination represented by a diamond shape in the figure. Each gene can be linked to N different variants which map on it. A digenic combination consists of two to four variants in two genes that determine the phenotype of a disease more clearly than the variant(s) in either gene alone. A combination can consist of 2 (di-allelic), 3 (tri-allelic) or 4 (tetra-allelic) different variants. Different digenic combinations can lead to the same disease.

DIDA implements a multi-tier architecture. At the lowest level of this architecture, the collected data are stored in a relational database based on the MySQL database management system (https://www.mysql.com). The relational database is structured in four tables representing the four main domain concepts or entities, which are genes, variants, digenic combinations and diseases (see Figure 1). Each table contains information and annotations representing properties or attributes of the corresponding entity. A detailed description of all the attributes provided by DIDA for each table can be found in the website documentation page (http://dida.ibsquare.be/documentation/).

Each entity is linked to the others through specific relationships as illustrated in Figure 1: A variant is mapped on a gene, a gene is indirectly linked to a digenic combination through the variants it contains, variant combinations (four alleles) in two genes form a digenic combination, a disease is caused by one or more digenic combinations. The central concept in DIDA is a digenic combination, which represents a true example of a patient with a specific digenic disease. Each patient has at least two variants in two different genes, named ‘digenic combination’, which together are causative for the patient's phenotype.

## WEB INTERFACE

At the higher level of the multi-tier architecture, DIDA implements a user-friendly web interface based on jQuery (https://jquery.com). The communication between the client-side layer and the server-side logic layer takes place through a communication protocol based on the JavaScript Object Notation (JSON) format. DIDA provides a web interface rich of content and functionalities allowing the user to retrieve all the information linked to a gene, variant, disease and digenic combination of interest. The website is organized in different pages providing browsing and search functionalities, as well as documentation and help pages, general database statistics and references to all the original papers from which the data have been collected. To encourage the participation of users, the website also provides the possibility to contribute novel data to DIDA. Through the Submit page (http://dida.ibsquare.be/submit/), the user can send information regarding (un)published digenic combinations which are not present in the database yet. A complete overview on how to use DIDA and its website can be found at http://dida.ibsquare.be/help/.

### Browsing, searching and downloading data

**Figure 2.**
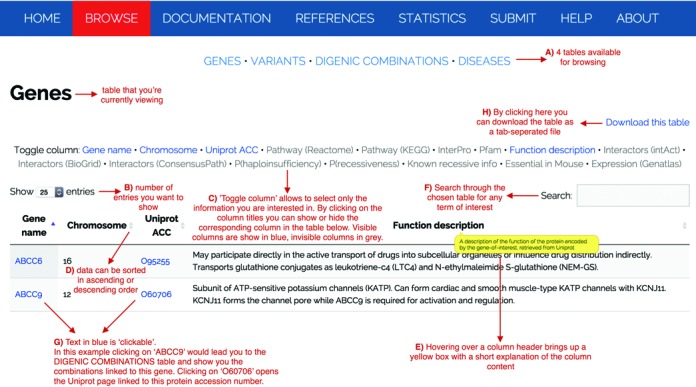
Snapshot of the Browse page and the genes table (http://dida.ibsquare.be/browse/).

DIDA provides different means for browsing and retrieving information from the database. The Browse page (http://dida.ibsquare.be/browse/) represents the entry point to four main tables containing information on genes, variants, digenic combinations and diseases, as shown in Figure 2(A). Figure 2 gives an overview of the table functionalities. The letters in brackets (from A to H) in the caption correspond to the letters reported in this figure. In each table view, the first 25 entries are shown by default, but the user can easily select the amount of entries to be visualized from a pop-up menu at the left-hand side and even decide to visualize all of them at once (B). Each table shows by default a certain amount of columns containing the data present in DIDA. ‘Toggle column’ allows the user to only visualize the columns of interest (C). Furthermore, all columns can be sorted in ascending or descending order (D) and hovering over a column header brings up a box with a short explanation of the column content (E). On the right-hand side, a search field allows the user to filter the visualized data according to textual criteria (F). For instance, the user may be interested in a specific gene or disease. Searching for a gene name will result in a search covering all the table columns, therefore, other genes that may be linked to the gene of interest (for instance because that gene name is mentioned in their functional description) may be included in the search results. By clicking on the gene name, the user has the possibility to browse also between different tables and look for a gene, variant or disease in the context of the digenic combinations (G). Therefore, the tables have a number of internal cross-links but also links to external resources as the ones already mentioned in the Annotation section. Tables and selected columns may be then directly downloaded in a tab-delimited format file by clicking on the link ‘Download this table’ on the top right-hand side of each table (H).

### A detailed view on a digenic combination

**Figure 3.**
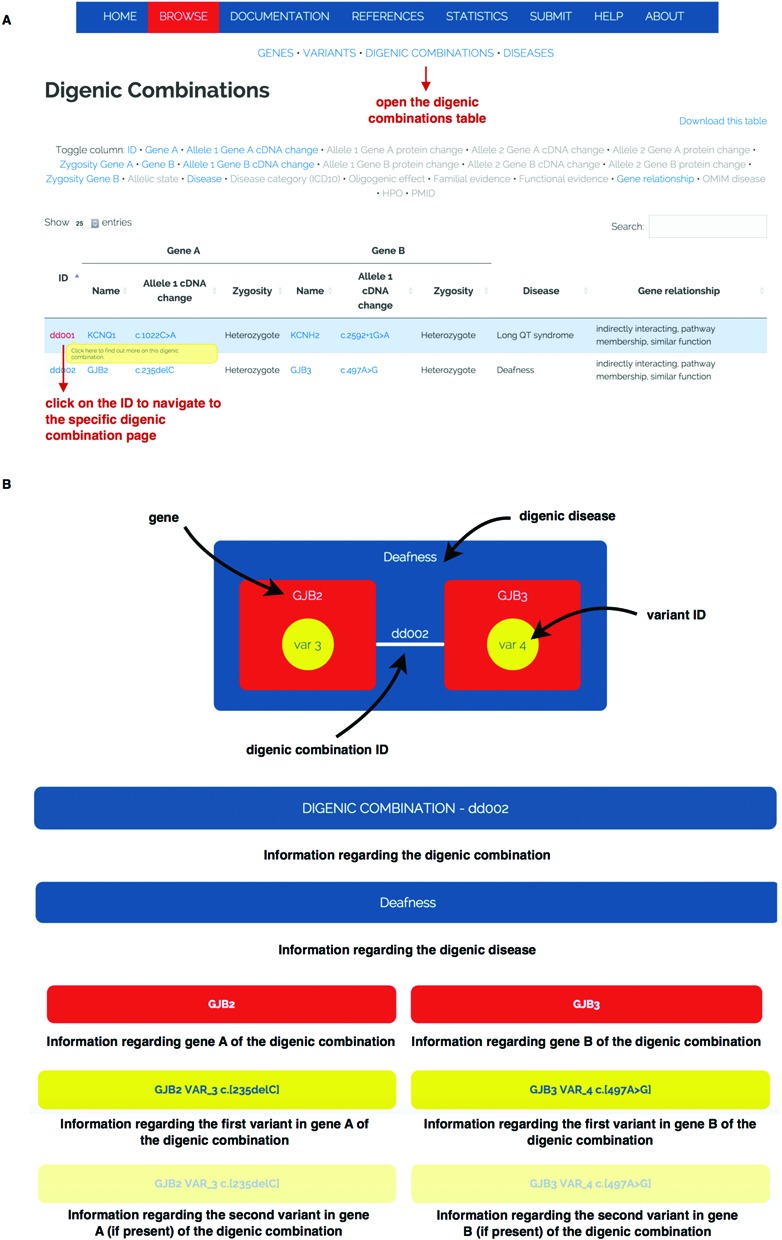
Panel A indicates how to navigate to the specific digenic combination page. Panel B gives a schematic description of a digenic combination example page. This page contains information on different levels ranging from the digenic combination annotations to the information regarding the two genes involved and the variants causative for the digenic disease.

DIDA also offers the possibility to look at the complete information linked to a digenic combination at once in a single page. By clicking on a digenic combination ID from the digenic combinations table (see Figure 3(A)), the user can surf to a detailed page describing the combination of interest. An example of how this page is organized is shown in Figure 3(B). The page shows on top, in pictorial format, the genes (in red) and variants (in yellow) involved in the digenic combination (white line) and the disease caused by the digenic combination (in blue). A hierarchy of descriptions follows. Detailed information about the specific digenic combination and the disease caused by this combination (blue headers) is first provided. This is followed by a description of the two genes involved in the combination (red headers) and finally the information on the variants is displayed below yellow headers.

## DATA STATISTICS

The current version of DIDA consists of 213 digenic combinations involved in 44 different diseases. These digenic combinations include 364 distinct variants, which are distributed over 136 distinct genes. Almost all variants are nonsynonymous: 68.41% are missense, 13.74% are frameshift and 8.79% are nonsense. All intronic (2.75%) and silent (0.82%) variants occur in combination with a nonsynonymous variant. The remaining 5.49% belong to either insertions/deletions or splicing variants (see also http://dida.ibsquare.be/statistics). Respectively, 80.22% and 72.8% of single variants are not reported in the 1000 genomes and ESP6500 projects. Furthermore, 16.21% (1000 genomes) and 23.9% (ESP6500, same value for European and African American populations) have a minor allele frequency of less than 1%, meaning that almost all variants in DIDA can be considered as unknown or rare variants.

A digenic combination is composed of four alleles unless one gene is on chromosome X and the patient is male or one gene is located in the mitochondrial genome, in which case there are three alleles. If two of those four alleles are different from the reference sequence, the digenic combination is considered as di-allelic. When there are three or four variant alleles, the combination is classified as respectively tri-allelic or tetra-allelic. Almost two-thirds (62.44%) of the digenic combinations present in DIDA are di-allelic. The other third (35.68%) are tri-allelic examples, with more than half belonging to one disease: Bardet-Biedl syndrome (13). There are also four tetra-allelic examples (1.88%) where the variants are present in a homozygous state. A 26.29% of digenic combinations belong to the ‘severity’ class, 31.92% are classified as ‘on/off’ and for 41.78% there was no classification to be derived based on the information present in the publication. The most represented digenic disease in DIDA is Bardet-Biedl syndrome (13), with almost 20% of digenic combinations (43 patients), 20% of variants and 9% of genes mapped to this disorder. Long QT syndrome (14) and Kallmann syndrome (16) are also well represented, with respectively 21 and 19 digenic combinations. It is not surprising that those three disorders form a large group in DIDA since their oligogenic nature is well studied. However, the majority (29 out of 44 or 66%) of diseases in DIDA are represented by only one or two digenic combinations, highlighting the need to focus on oligogenic inheritance. Our analysis also reveals that for more than half of the digenic combinations there is familial or functional evidence supporting the digenic status of the disease in the affected patient. Thirteen out of 213 (6.1%) examples contain evidence at both levels. A visualization of all the statistics can be found via http://dida.ibsquare.be/statistics.

Essential to digenic, and more general oligogenic diseases, is the relationship that exists between the genes involved in the disease. Literature has shown that the genes that cause a digenic disease often have a physical or functional relationship (5,19). For each digenic combination in DIDA we determined this relationship, focussing on five different relationship types, based on the properties of the genes and/or proteins they encode (see Table 1):

- *Directly interacting* genes relate digenic combinations for which the two genes are annotated as directly interacting in BioGrid (36), IntAct (35) or ConsensusPathDb (37).
- *Indirectly interacting* genes are the digenic combinations for which the two genes share a third gene with whom they directly interact according to BioGrid (36), IntAct (35) or ConsensusPathDb (37).
- *Pathway membership* refers to genes in a digenic combination that have the same pathway annotation in KEGG (30) or Reactome (31).
- *Co-expression refers* to digenic combinations for which the two genes are transcribed in the same tissue(s) according to the annotations retrieved from GNF/Atlas (38).
- *Similar function* pairs are those in which the two genes have in common one or more functionally conserved motifs or conserved domains in Pfam (32) or InterPro (33).

The 213 digenic combinations present in DIDA are composed of 116 (54.46%) unique gene pairs. Twenty-one of the latter (18.10%) cannot be classified in either one of the above five relationship categories, 30 (25.86%) show one type of relationship and more than half (65 or 56.03%) belong to multiple categories. Table 1 reports on the statistics for each molecular mechanism.

**Table 1.** Number and percentage of relationships between pairs of genes carrying variants causative for the digenic disease. Five different relationship types are defined

Type of molecular mechanism	Number (%) of unique gene
for gene pairs	pairs with this type of molecular
	mechanism
Directly interacting	40 (34.48%)
Indirectly interacting	69 (59.48%)
Pathway membership	25 (21.55%)
Co-expression (RNA)	48 (41.38%)
Similar function	17 (14.66%)

## DISCUSSION

DIDA provides a first comprehensive resource for digenic diseases, integrating at this moment information related to 134 genes, 364 variants, 213 digenic combinations and 44 diseases originating from 82 publications. Notwithstanding the exclusion of some publications due to the lack of sufficient information or because one or both loci were a CNV (or repeat), DIDA covers almost all of the digenic examples present in literature until June 2015. As argued in (19), substantial proof for digenic inheritance is present when there is evidence of protein–protein or protein–DNA interaction for the two genes or proteins, when there is segregation of the digenic combination with the phenotype in the family and/or when there is a combined effect of the variants at the functional level. For each digenic combination, the absence or presence of familial and functional evidence was extracted from the corresponding publication. For more than half of the digenic combinations, there was evidence supporting digenicity at one level, only a minority contained evidence at both levels. Trying to fill this gap, the analysis leading up to Table 1 showed that 81.9% of unique gene pairs display at least one type of relationship. Taking into account all gene relationships, familial and functional evidence for digenic inheritance, there are 28 (13.15%) digenic combinations that are not supported by any of these types, meaning that the digenic status of these examples should be treated with caution. However most of the digenic combinations present in DIDA show either one type (87 or 40.85%) or two types (86 or 40.38%) of evidence. Twelve digenic combinations (5.63%) are even supported by all three types of evidence meaning that for these examples there is substantial proof for digenic inheritance.

Researchers can themselves submit their (un)published work for consideration via http://dida.ibsquare.be/submit/. The prerequisites and an interactive submission form are present at this page. For now only SNVs and short indels up to length 50 will be accepted. There must be evidence for the digenic status of the disease present in the patient, meaning that the variant genotypes at two loci explain the phenotype of the patient more clearly than the genotypes at one locus alone. The user is encouraged to submit information using standardized terminology (e.g., Orphanet disease name, HPO terms to describe the patient's phenotype, gene names following HGNC nomenclature). The attributes present in the DIDA database, that are absent from the submission form, will be automatically retrieved from the corresponding databases by the DIDA staff.

In conclusion, DIDA provides a first manually curated repository on digenic diseases. It will serve as a benchmark dataset used for the development of new bioinformatic tools. Furthermore, DIDA provides an important tool for clinical and molecular geneticists to find the most comprehensive information on the digenic nature of their diseases of interest. It provides detailed phenotypical information about the digenic case that can give the geneticist a better insight into the specific disease. Next to updating the database with data on new digenic instances, we plan to extend DIDA with other variant types like large indels and CNVs in the future. Moreover, we are convinced that the initial version of DIDA and future updates will provide a valuable resource for understanding digenic diseases, which we hope will be exploited by different research communities.
